# Comparative transcriptome revealed the molecular responses of *Aconitum carmichaelii* Debx. to downy mildew at different stages of disease development

**DOI:** 10.1186/s12870-024-05048-x

**Published:** 2024-04-25

**Authors:** Lijuan Chen, Yiwen Hu, Li Huang, Long Chen, Xianglei Duan, Guangzhi Wang, Hong Ou

**Affiliations:** 1 State Key Laboratory of Southwestern Chinese Medicine Resources, Chengdu University of Traditional Chinese Medicine, Chengdu, 611137 China; 2 College of Pharmacy, Chengdu University of Traditional Chinese Medicine, Chengdu, 611137 China; 3 Hospital of Chengdu University of Traditional Chinese Medicine, Chengdu, 610072 China

**Keywords:** *Aconitum carmichaelii*, Downy mildew, Responses, Transcriptomic analysis, Plant hormones

## Abstract

**Background:**

*Aconitum carmichaelii* Debx. has been widely used as a traditional medicinal herb for a long history in China. It is highly susceptible to various dangerous diseases during the cultivation process. Downy mildew is the most serious leaf disease of *A. carmichaelii*, affecting plant growth and ultimately leading to a reduction in yield*.* To better understand the response mechanism of *A. carmichaelii* leaves subjected to downy mildew, the contents of endogenous plant hormones as well as transcriptome sequencing were analyzed at five different infected stages.

**Results:**

The content of 3-indoleacetic acid, abscisic acid, salicylic acid and jasmonic acid has changed significantly in *A. carmichaelii* leaves with the development of downy mildew, and related synthetic genes such as 9-cis-epoxycarotenoid dioxygenase and phenylalanine ammonia lyase were also significant for disease responses. The transcriptomic data indicated that the differentially expressed genes were primarily associated with plant hormone signal transduction, plant-pathogen interaction, the mitogen-activated protein kinase signaling pathway in plants, and phenylpropanoid biosynthesis. Many of these genes also showed potential functions for resisting downy mildew. Through weighted gene co-expression network analysis, the hub genes and genes that have high connectivity to them were identified, which could participate in plant immune responses.

**Conclusions:**

In this study, we elucidated the response and potential genes of *A. carmichaelii* to downy mildew, and observed the changes of endogenous hormones content at different infection stages, so as to contribute to the further screening and identification of genes involved in the defense of downy mildew.

**Supplementary Information:**

The online version contains supplementary material available at 10.1186/s12870-024-05048-x.

## Introduction

*Aconitum carmichaelii* Debx. is a medicinal plant in Ranunculaceae family. Its lateral and mother roots have long been used as medicine, which are known as “Fuzi” and “Chuanwu” respectively [1]. Meanwhile, the leaves of *A. carmichaelii* also have certain pharmacological effects that can produce economic value [2]. The characteristic aconite alkaloids in *A. carmichaelii* roots have multiple pharmacological activities such as analgesic, anti-inflammatory, local anesthesia, anti-cancer, etc. [3, 4]. It is mainly used for neurological diseases [5], rheumatic diseases [6], tumors, shock [7] and various pain symptoms in clinical treatment. *A. carmichaelii* has a long planting history of over one thousand years in China. It is mainly distributed in Sichuan, Yunnan and Shaanxi provinces [8] in China, with Jiangyou in Sichuan being the most authentic one. Presently, cultivation is the primary measure employed for obtaining medicinal resources. There are numerous diseases occur in the planting, such as root rot [9], leaf spot and downy mildew. These diseases often result in mixed infections, which can reduce yields by over 50 percent and even cause devastating economic losses in severe cases. Downy mildew is a leaf disease with high prevalence and strong infectivity in the Fuzi planting base in Jiangyou, which usually occurs and spreads from March to May. There are short of effective control methods yet. The pathogen of *A. carmichaelii* downy mildew (ADM), caused by *Peronospora aconiti*, induces a mold layer on the back of the leaves and causes dwarf plants and reduced production. *P. aconiti* is a special obligate parasite of the *Peronospora* in Peronosporaceae, and there are still limited researches on ADM owing to the difficulty in separation and culture. The research on ADM is not systematic enough, however, an understanding of the response mechanism of *A. carmichaelii* to ADM is urgent for disease control.

Plant pathogens are a form of biological stress that typically cause abnormal growth and dysfunction in plants. More and more researchers are focusing on the interaction mechanisms between pathogens and their host plants. The response of plants to pathogen infection is an important aspect of plant-pathogen interactions and is also crucial for the study of plant defense. RNA sequencing (RNA-seq), a modern technology for transcriptomic profiling, has been widely applied to identify functional pathways and responsive genes in response to pathogen stress. Rajarammohan [10] identified the differentially expressed genes (DEGs) in *Brassica juncea* associated with the pathogenicity of *Alternaria brassicae* via transcriptome sequencing. Based on the comparative transcriptomic analysis of *Cucumis melo* powdery mildew infection between a disease-resistant cultivar and a disease-susceptible cultivar, Zhao [11] conducted identification of DEGs and enrichment analysis, and ultimately speculated on the main disease-resistant regulatory pathways including the xyloglucan metabolic process, response to oxidative stress and hydrolase activity. RNA-seq can help us understand the host–pathogen interactions. Previously the transcriptome analysis of two leaf-type cultivars of *A. carmichaelii* speculated the correlation between leaf type and root rot resistance [12]. However, the research on the response mechanism of *A. carmichaelii* downy mildew has not yet been go ahead.

Plant endogenous hormones are regulatory factors of plant immunity and play an important role in plant disease responses [13]. Plant hormones and the signal regulation of them can affect the resistance of the host and the pathogenicity of the pathogen, which are helpful for controlling plant diseases [14, 15]. In this study, the transcriptomic analysis of *A. carmichaelii* leaves at differential ADM stages was performed, and the DEGs involved in response to ADM were annotated. Hub genes were identified by constructing a weighted gene co-expression network analysis (WGCNA). Meanwhile, the mechanism plant hormones regulated ADM were revealed by the changes in plant hormone contents and signaling pathways. The results also provided insights into the molecular defense mechanisms of *A. carmichaelii*, and provided deeper thoughts on the further breeding and application of resistance to ADM.

## Materials and methods

### Plant materials

In May 2022, the *A. carmichaelii* leaves used in this study were collected from the Jiangyou Planting Base (31°43′56" N, 104°42′2" E) in Jiangyou County, Mianyang City, Sichuan Province, China. Leaves infected by *P. aconiti* in same natural living conditions were collected at disease stages 0 to 4 (named as JH, JO, JW, JT and JF, shown in Table 1), and the collected leaves were all from the second node of stems of plants with similar growth status. Three leaves with consistent growth and disease symptoms were selected and mixed into one sample. Four biological replicates were then frozen separately in liquid nitrogen and stored at -80 ℃ until further experiments.

**Table 1:** Grading criteria for downy mildew of *A. carmichaelii*

Name	Stage	Disease symptoms	Group
JH	Disease stage 0	Healthy leaf	Control group
JO	Disease stage 1	Infected leaf area < 1/4	Disease group 1
JW	Disease stage 2	1/4 < Infected leaf area < 1/2	Disease group 2
JT	Disease stage 3	1/2 < Infected leaf area < 3/4	Disease group 3
JF	Disease stage 4	Infected leaf area > 3/4	Disease group 4

### Plant hormones contents determination

The content of endogenous hormones, including indole-3-acetic acid (IAA), abscisic acid (ABA), salicylic acid (SA) and jasmonic acid (JA), was determined using Enzyme-Linked ImmunoSorbent Assay (ELISA) Kits (Meimian, Jiangsu, China).

### RNA preparation and RNA-seq

Total RNA was extracted using the RNAprep Pure Plant Plus Kit (TIANGEN, Beijing, China) according to the manufacturer’s procedures. The quantity and purity of total RNA were analyzed by Bioanalyzer 2100 and RNA 1000 Nano LabChip Kit (Agilent, CA, USA) (RIN ≥ 7.0, OD_260/280_ = 1.8–2.2, OD_260/230_ = 1.8–2.2). Poly-A RNA was purified from the total RNA using Oligo (dT) magnetic beads through two rounds of purification. After purification, the mRNA was fragmented into small-pieces using divalent cations at an elevated temperature. Then the cleaved RNA fragments were reverse-transcribed to create the final cDNA library, the average insert size for the paired-end-libraries was 300 bp. The paired-end sequencing were performed using the Illumina Novaseq™ 6000 platform (LC Sceiences, USA).

### Assembly and gene expression analysis

High-quality reads were obtained with Cutadapt v1.9 by removing low-quality bases, adapter-contaminated bases, and undetermined bases [16]. The de novo assembly of the transcriptome was performed with Trinity v2.4.0 to obtain unigenes [17]. All assembled unigenes were aligned against the gene ontology (GO), KEGG, NR, SwissProt, Pfam and eggNOG databases (E ≤ 1e-5) [18]. Transcripts per kilobase million (TPM) were used as an indicator to measure unigenes expression levels [19]. The DEGs between the two groups was analyzed using DESeq2 [20]. *P*-value ≤ 0.05 and |log2FC|≥ 1 were used as the thresholds for significant DEGs.

### WGCNA

WGCNA was performed using WGCNA in the R package [21]. Genes with an FPKM < 1 in any sample were filtered out. The relationship of connectivity of genes was obtained using the Omicsmart online software. Co-expression relationships in candidate functional modules were analyzed and visualized with Cytoscape v3.9.0 [22].

### Real-time quantitative polymerase chain reaction

To validate the RNA-seq, eight DEGs were randomly selected for performing real-time quantitative polymerase chain reaction (qRT-PCR). Total RNA was used for cDNA synthesis with the RT Easy™ II (with gDNase) (Foregene, Chengdu, China) according to the manufacturer’s instructions. The 20 µL qRT-PCR reaction system contained 10 μL 2 × Real PCR Easy™ Mix-SYBR (Foregene, Chengdu, China), 0.8 μL each of forward and reverse primers, 5 μL cDNA template, and 3.4 μL double-distilled H_2_O. The reaction procedure using the SYBR green method was as follows: preheat at 95 ℃ for 3 min, then perform 40 cycles of denaturation at 95 ℃ for 5 s and anneal at 60 ℃ for 30 s. We used the eukaryotic initiation factor (EIF) from the transcriptome data of this study as the reference gene [23]. The primers used were listed in Table S1. The relative expressions were calculated using the 2^−∆∆Ct^ method, and the Log2FC was calculated by comparing the four disease groups (JO, JW, JT, JF) with the control group (JH).

### Transcription factors prediction

The online transcription factor (TF) prediction software (PlantTFDB, http://planttfdb.cbi.pku.edu.cn/prediction.php) [24] was used to predict the TFs of DEGs.

## Results

### Disease symptoms of ADM

**Fig. 1:**
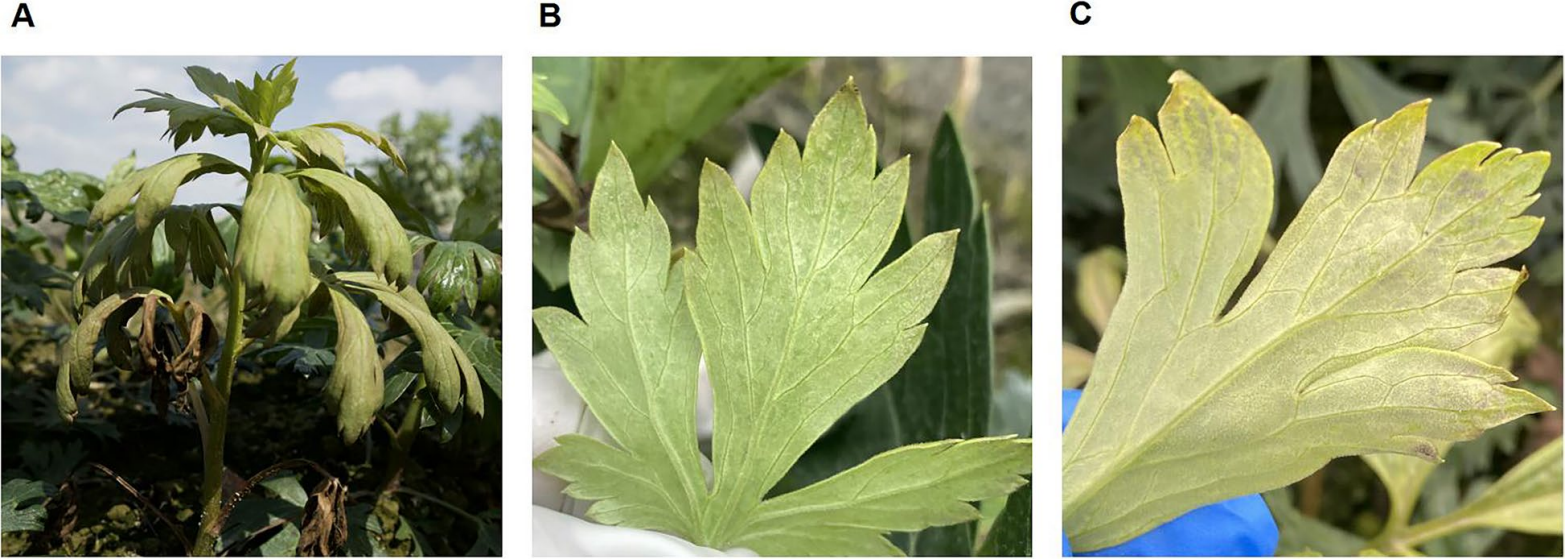
Disease symptoms of ADM. **A** Infected plant. **B** Infected leaf at ADM early stage. **C** Infected leaf at ADM late stage

After being infected with ADM, the leaves of *A. carmichaelii* initially displayed a curling effect along with a changing color from emerald green to grayish-green. Then the leaves began to thicken and curl downward and inward, while a gray-white mold layer was formed on the back of the leaves simultaneously. Different stages of ADM leaves exhibited varying degrees of chlorosis and curling (Fig. 1A). The field observations showed that a gray-white mold layer appeared on the back edge of the leaves during disease stage 1 (Fig. 1B). As ADM worsened, the area of the mold layer increased, causing the leaves to gradually turn yellow. At the disease stage 4, the back of the leaves was already densely covered with a gray-brown mold layer, and even the entire plant showed signs of drying (Fig. 1C).

### Plant hormones contents

**Fig. 2:**
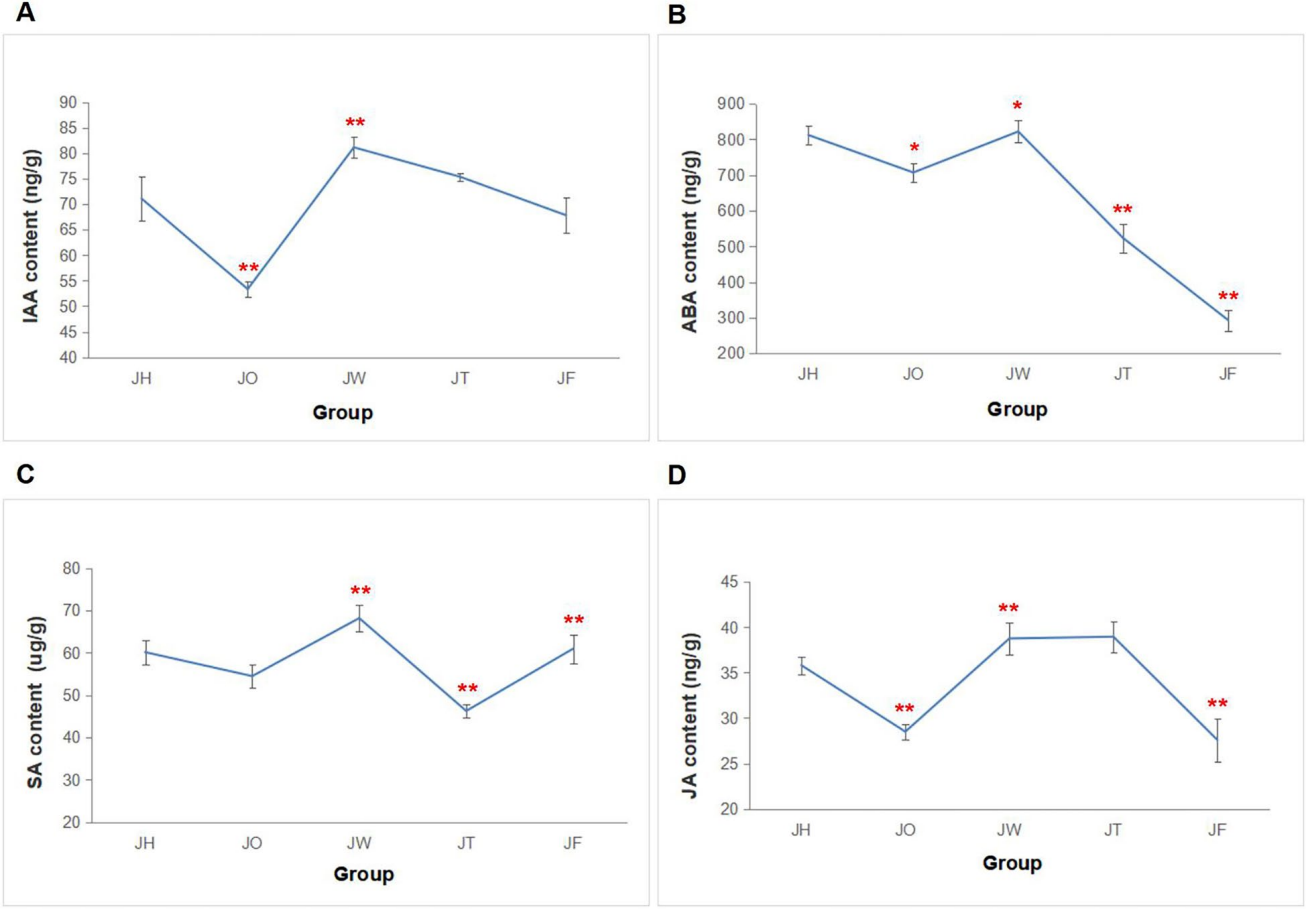
Plant hormones contents of *A. carmichaelii* leaves at different ADM stages. **A** IAA content. **B** ABA content. **C** SA content. **D** JA content. (* and ** represent signifcant diferences at *p* < 0.05 and *p* < 0.01, respectively between the two sets of data)

There were significant changes in the content of endogenous hormones at different disease stages (*P* ≤ 0.05) (Fig. 2). The contents of IAA and ABA were initially significantly reduced in JO compared to JH, followed by a significant increase to their highest levels in JW. IAA content was a gradual decrease in JT and JF, while ABA content was significantly decreased in JT and JF. The contents of SA and JA were initially decreased in JO before obviously increasing to their highest levels in JW. SA content was eventually significantly increased again in JF after having been reduced markedly in JT. However, JA experienced an obvious decrease in JF following an increase in JT. During the disease stage 3 and 4, SA and JA exhibited contrasting changes.

### RNA-seq quality

Five disease stages (JH, JO, JW, JT and JF) were selected as sampling points for RNA-seq to collect a sufficient number of transcripts. A total of 20 samples were sequenced, and a further 128.33 Gb of raw data and 115.84 Gb of clean reads were obtained. Each sample contained more than 5 Gb of clean data. The ratios of Q20 and Q30 were higher than 96% and 90% respectively, with a GC content ranging from 45.01% to 48.67% (Table S2). The transcriptomic sequencing was successful and met the necessary requirements for further analysis.

### Assembly statistics

The total number of transcripts identified for de novo transcriptome assembly was 225,562, with an N50 value of 1,175 bp, a median length of 542 bp, and a maximal length of 20,656 bp. And 85,300 unigenes were obtained with an N50 value of 1,319 bp, a median length of 581 bp, and the same maximal length with transcripts. The length distribution of the transcriptome was analyzed and all the assembled transcripts and unigenes were exceeded 200 bp. Among them, transcripts and unigenes between 200 ~ 300 bp were the largest proportion, accounting for 21.98% and 22.88%, respectively (Figs. S1 and S2). The average GC contents in the transcripts and unigenes were 43.72% and 44.61%, respectively.

### Gene expression changes in different groups

To identify the differences in gene expression between various ADM stages, we compared the DEGs among the four groups (JO vs JH, JW vs JH, JT vs JH and JF vs JH). For each group, the number of up-regulated genes was 10,331, 9,829, 10,058 and 4,145, respectively, and the number of down-regulated genes was 3,315, 2,769, 2,342, and 2,456 (Fig. S3). This suggested that *A. carmichaelii* exhibited more significant transcriptional alterations in the initial phase of downy mildew. Meanwhile, the overall number of up-regulated DEGs was more than that of down-regulated DEGs, indicating that most DEGs were activated while the few were suppressed after ADM.

### Functional classifications and pathway analyses of the DEGs

**Fig. 3:**
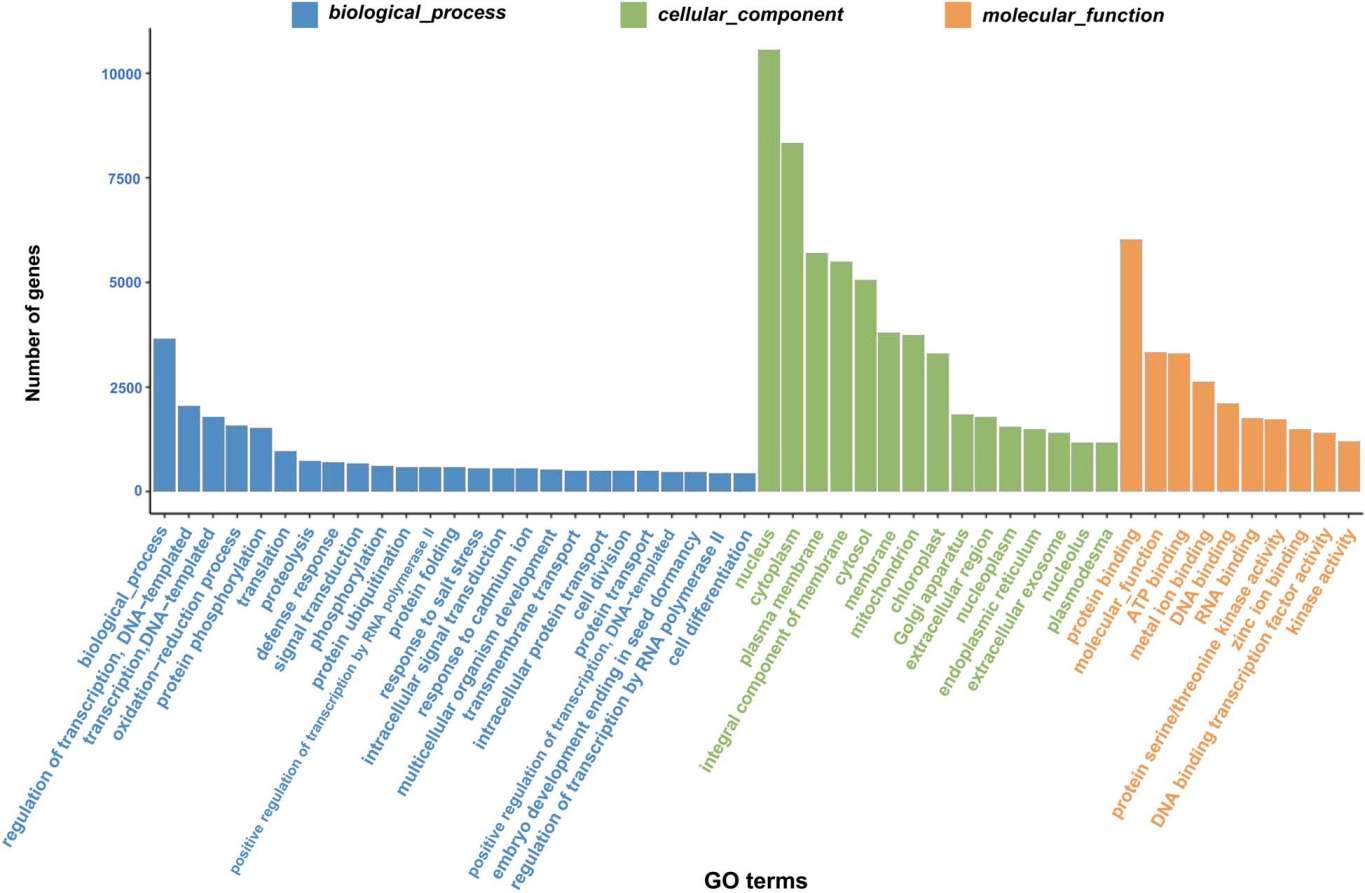
GO enrichment analysis of DEGs

To determine the biological functions of the DEGs in different groups, GO and KEGG enrichment analyses were conducted. The GO enrichment analysis showed that the DEGs involved in responding to ADM were enriched in the cellular components (CC), biological processes (BP) and molecular functions (MF). In total, 42,902 genes were annotated in the GO database. Among the JO vs JH, JW vs JH, JT vs JH and JF vs JH comparisons, the most abundant term in BP classification was biological process (GO:0008150) such as DNA-templated transcription (GO:0006351), regulation of DNA-templated transcription (GO:0006355), protein phosphorylation (GO:0006468), and oxidation–reduction process (GO:0055114). In contrast, those in MF classification included protein binding (GO:0005515), molecular function (GO:0003674), ATP binding (GO:0005524) and metal ion binding (GO:0046872), and those in the CC classification included nucleus (GO:0005634), cytoplasm (GO:0005737), plasma membrane (GO:0005886) and integral component of membrane (GO:0016021) (Fig. 3).

**Fig. 4:**
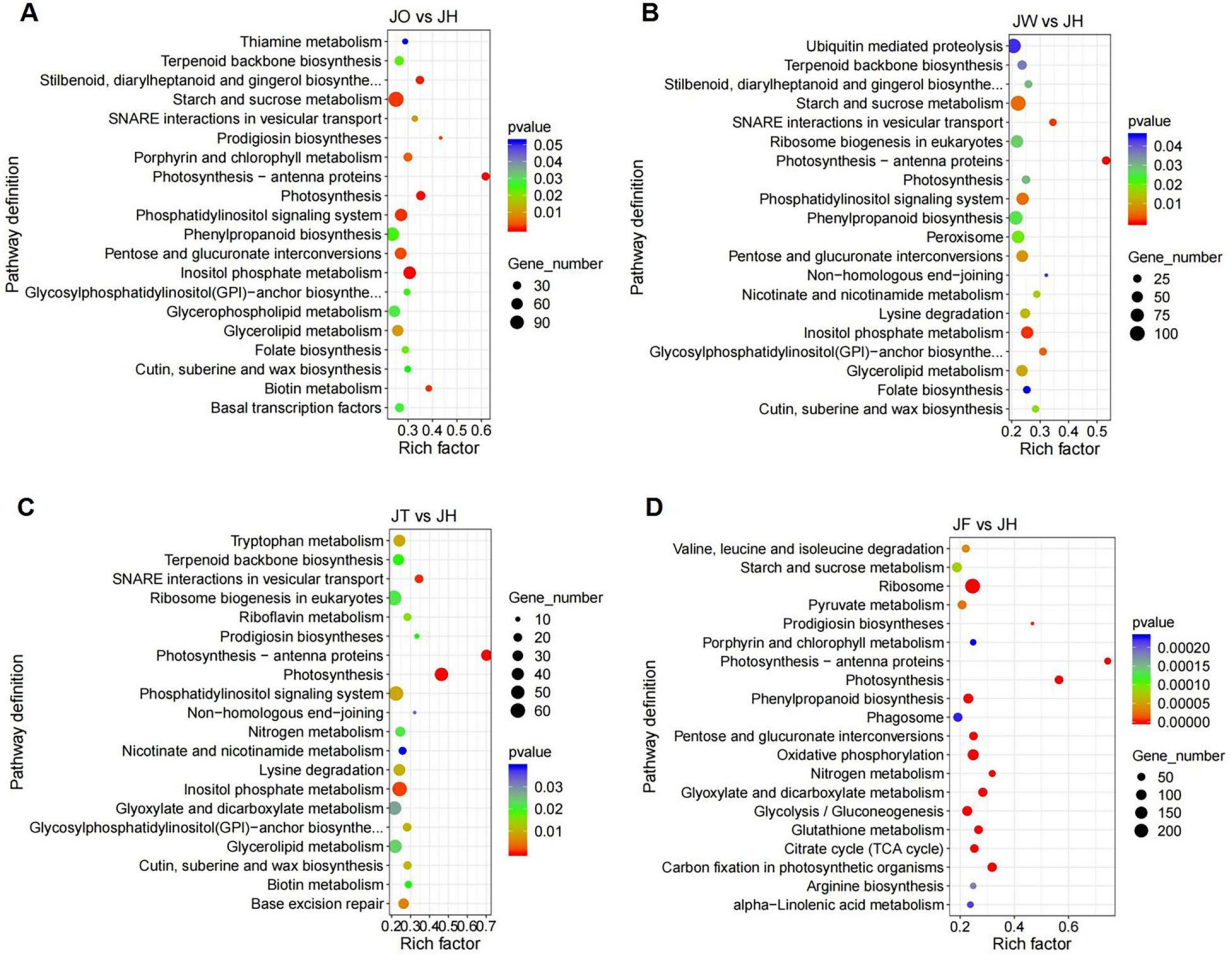
KEGG pathway enrichment analysis of DEGs. **A** KEGG annotation of DEGs in JO vs JH. **B** KEGG annotation of DEGs in JW vs JH. **C** KEGG annotation of DEGs in JT vs JH. **D** KEGG annotation of DEGs in JF vs JH

In total, 29,784 DEGs were annotated in the KEGG database, and those of the four disease stages were annotated to 134, 136, 133 and 132 pathways, respectively. The mostly distributed pathways were plant hormone signal transduction (ko04075), plant-pathogen interaction (ko04626), mitogen-activated protein kinase (MAPK) signaling pathway-plant (ko04016), phenylpropanoid biosynthesis (ko00940) and protein processing in the endoplasmic reticulum (ko04141) (sorted according to the number of annotated genes) (Table S3). The DEGs of JO vs JH were mainly involved in spliceosome (ko03040), protein processing in endoplasmic reticulum (ko04141) and starch and sucrose metabolism (ko00500). The DEGs of JW vs JH were primarily associated with the spliceosome, purine metabolism (ko00230) and starch and sucrose metabolism. The DEGs of JT vs JH were predominantly associated with the spliceosome, purine metabolism, and pyrimidine metabolism (ko00620). The DEGs of JF vs JH were mainly enriched in ribosomes (ko03010), protein processing in the endoplasmic reticulum and oxidative phosphorylation (ko00190). In addition, photosynthesis (ko00195) and photosynthetic antenna proteins (ko00196) were the most notably enriched pathways during all disease stages (Fig. 4A-D).

### Functional identification of DEGs

The analysis of KEGG enrichment pathways indicated that there are abundant metabolic pathways at different ADM stages. We primarily examined three pathways associated with disease responses: plant-pathogen interactions, MAPK signaling pathway in plants, and phenylpropanoid biosynthesis pathway. The plant-pathogen interaction pathway was enriched with 70, 70, 63, and 73 DEGs in JO vs JH, JW vs JH, JT vs JH and JF vs JH, respectively. The DEGs were mostly up-regulated at all four disease stages, including 53 up-regulated DEGs in JO vs JH, 46 up-regulated DEGs in JW vs JH, 49 up-regulated DEGs in JT vs JH and 53 up-regulated DEGs in JF vs JH (Table S4). In the fungal PAMP pathway, 13, 9, 12 and 5 DEGs encoding calcium-dependent protein kinases (CDPK) were identified in JO vs JH, JW vs JH, JT vs JH, and JF vs JH, respectively. And there were 1, 3, 3 and 2 DEGs encoding respiratory burst oxidase homologue (RBOH) including 1, 1, 2 and 2 up-regulated DEGs. Similarly, 6, 6, 7 and 10 DEGs involved in the regulation of calmodulin (CAM), and 7, 10, 7 and 12 DEGs regulated calcium-binding proteins (CML). Most of them were up-regulated. In addition, 2 CML genes (TRINITY_DN45210_c4_g1, TRINITY_DN53694_c1_g1), 2 disease resistance protein RPM1 genes (TRINITY_DN47666_c2_g1, TRINITY_DN55634_c1_g2), 2 WRKY transcription factor 1 (WRKY1) genes (TRINITY_DN52457_c0_g1, TRINITY_DN52457_c0_g2) and 3 WRKY2 genes (TRINITY_DN54802_c2_g1, TRINITY_DN55902_c4_g1, TRINITY_DN60274_c0_g3) were significantly up-regulated at different ADM stages (Table S5-S8).

The MAPK signaling pathway was enriched with 93, 86, 88, and 62 DEGs in JO vs JH, JW vs JH, JT vs JH and JF vs JH, respectively. The DEGs were mostly up-regulated at all four disease stages, including 74 up-regulated DEGs in JO vs JH, 63 up-regulated DEGs in JW vs JH, 68 up-regulated DEGs in JT vs JH and 42 up-regulated DEGs in JF vs JH (Table S4). The MAPK signaling pathway interacts with plant hormone signaling pathways and plant-pathogen interactions, such as the flg22 pathway and ABA, JA and ethylene (ET) pathways. Defensin-like protein 16 (PDF 1.2) was regulated by 2 up-regulated genes in JO vs JH, 2 up-regulated genes and 1 down-regulated gene in JW vs JH, 4 up-regulated genes in JT vs JH, and 2 up-regulated genes and 2 down-regulated genes in JF vs JH. And there were 2 PDF 1.2 genes (TRINITY_DN38763_c1_g3, TRINITY_DN38774_c0_g1) were significantly up-regulated at all disease stages (Table S5-S8).

The phenylpropanoid biosynthesis pathway was enriched with 90, 82, 70, and 88 DEGs in JO vs JH, JW vs JH, JT vs JH and JF vs JH, respectively. The DEGs were mostly up-regulated at all four disease stages. This included 50 up-regulated DEGs in JO vs JH, 51 up-regulated DEGs in JW vs JH, 44 up-regulated DEGs in JT vs JH and 57 up-regulated DEGs in JF vs JH (Table S4). The DEGs encoding the phenylalanine ammonia lyase (PAL) were up-regulated at all stages of ADM, with 4 significantly up-regulated PAL genes (TRINITY_DN41665_c2_g4, TRINITY_DN41931_c1_g1, TRINITY_DN41931_c1_g3, TRINITY_DN49908_c0_g1) (Table S5-S8).

### Analysis of DEGs related to plant hormones

**Fig. 5:**
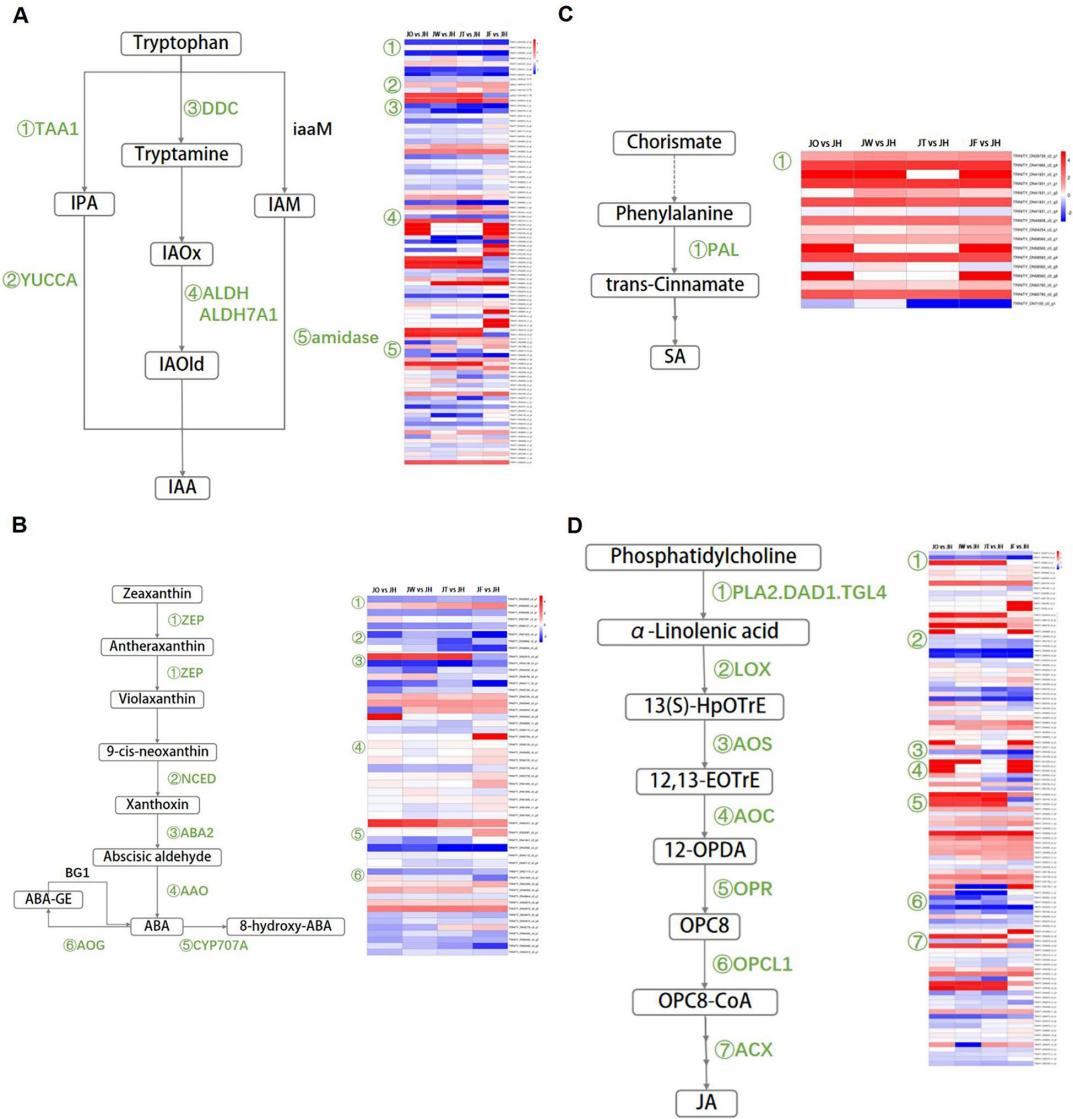
DEGs in plant hormone biosynthetic pathways. **A** AUX biosynthetic pathway and expression of related genes. **B** ABA biosynthetic pathway and expression of related genes. **C** SA biosynthetic pathway and expression of related genes. **D** JA biosynthetic pathway and expression of related genes. All images were granted permission by KEGG

With the transcriptomic data of *A. carmichaelii*, 95 DEGs were annotated to the auxin (AUX) biosynthesis pathway, including 7 L-tryptophan-pyruvate aminotransferase (TAA1) genes, 4 indole-3-pyruvate monooxygenase YUCCA (YUC) genes, 23 aromatic-L-amino-acid decarboxylase (DDC) genes, 25 aldehyde dehydrogenase (ALDH) genes, 7 aldehydede hydrogenase family 7 member A1 (ALDH7A1) genes and 29 amidase genes (Fig. 5A, Table S9). A total of 51 DEGs were involved in the ABA biosynthesis pathway, including 5 zeaxanthin epoxidase (ZEP) genes, 3 9-cis-epoxycarotenoid dioxygenase (NCED) genes, 13 xanthoxin dehydrogenase (ABA2) genes, 11 abscisic-aldehyde oxidase (AAO) genes, 5 ABA 8'-hydroxylase CYP707A (CYP707A) genes and 14 abscisate beta-glucosyltransferase (AOG) genes. All of the genes encoding NCED were down-regulated, and most of the AAO genes were relatively highly expressed in JF vs JH (Fig. 5B, Table S10). The genes involved in SA biosynthesis included only 17 PAL genes, and most of them were up-regulated and had a higher expression level in JW vs JH (Fig. 5C, Table S11). In the JA biosynthesis pathway, 106 DEGs were involved, including 2 phospholipase A2 (PLA2) genes, 10 phospholipase A1 (PLA1) genes, 3 triacylglycerol lipase TGL4 (TGL4) genes, 23 lipoxygenase (LOX) genes, 4 allene oxide synthase (AOS) genes, 7 allene oxide cyclase (AOC) genes, 20 12-oxophytodienoic acid reductase (OPR) genes, 8 OPC-8:0 CoA ligase 1 (OPCL1) genes and 29 acyl-CoA oxidase (ACX) genes (Fig. 5D, Table S12).

**Fig. 6:**
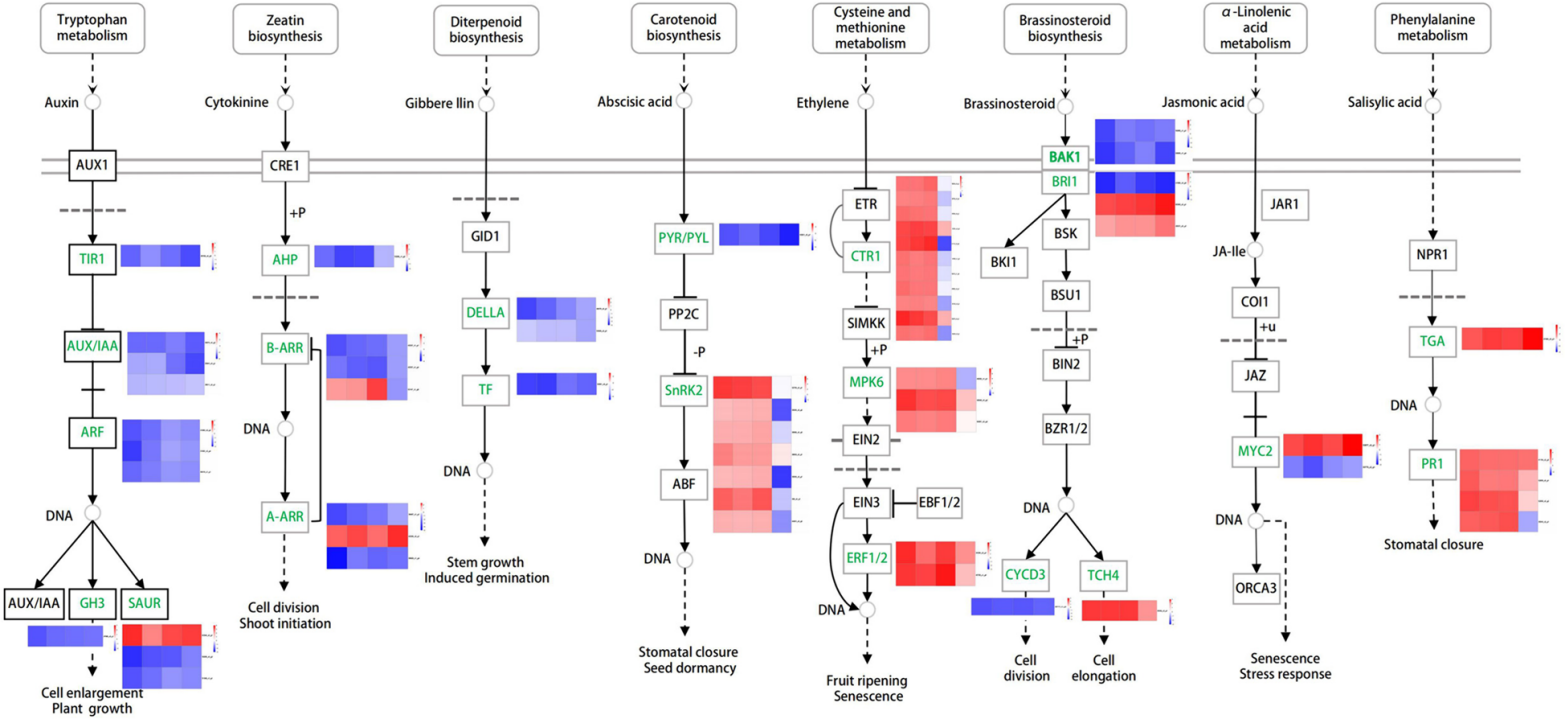
DEGs in plant hormone signal transduction pathway. This image was granted permission by KEGG

The plant hormone signal transduction pathway enriched 90, 74, 60, and 61 DEGs in JO vs JH, JW vs JH, JT vs JH and JF vs JH, respectively. The DEGs were mostly up-regulated at all four disease stages. This included 49 up-regulated DEGs in JO vs JH, 41 up-regulated DEGs in JW vs JH, 39 up-regulated DEGs in JT vs JH and 32 up-regulated DEGs in JF vs JH (Table S4). Many phytohormones play crucial regulatory roles in plant-pathogen interactions, such as ABA, SA, JA, AUX, cytokinin (CTK), gibberellin (GA), brassionosteroid (BR) and ET [25]. The DEGs that showed expressive changes at least three disease stages across multiple hormone signaling pathways were analyzed in the infected leaves of *A. carmichaelii* (Fig. 6). Some DEGs involved in the AUX signaling pathway have differential expression, including transport inhibitor response 1 (TIR1), AUX-responsive protein AUX/IAA (AUX/IAA), AUX-responsive factor ARF (ARF), SAUR family protein (SAUR) and AUX-responsive GH3 (GH3), which showed notably down-regulated expression. Most DEGs in the CTK signaling pathway, including two-component response regulators of the ARR family (ARR-A/ARR-B) and histidine-containing phosphotransfer peotein (HP), were down-regulated. All DEGs in the GA signaling pathway were down-regulated throughout the entire ADM process. Thus, the AUX, CTK and GA signaling pathways were inhibited after ADM infection. The serine/threonine-protein kinase SnRK2 (SnRK2) genes involved in the ABA signaling pathway were up-regulated at disease stage 1, 2, 3, and were down-regulated or slightly up-regulated at disease stage 4. In the ET signaling pathway, the genes encoding ET-responsive transcription factor 1 (ERF1) were up-regulated at each stage and serine/threonine-protein kinase constitutive triple response 1 (CTR1) and mitogen-activated protein kinase 6 (MPK6) were up-regulated throughout the entire disease process, with the exception of disease stage 4. In brief, the expression levels of genes related to ABA and ET signaling pathways were lower in disease stage 4 than in other disease stages. The DEGs in the SA signaling pathway were mainly up-regulated (Fig. 6, Table S5-S8).

### Candidate genes based on WGCNA

**Fig. 7:**
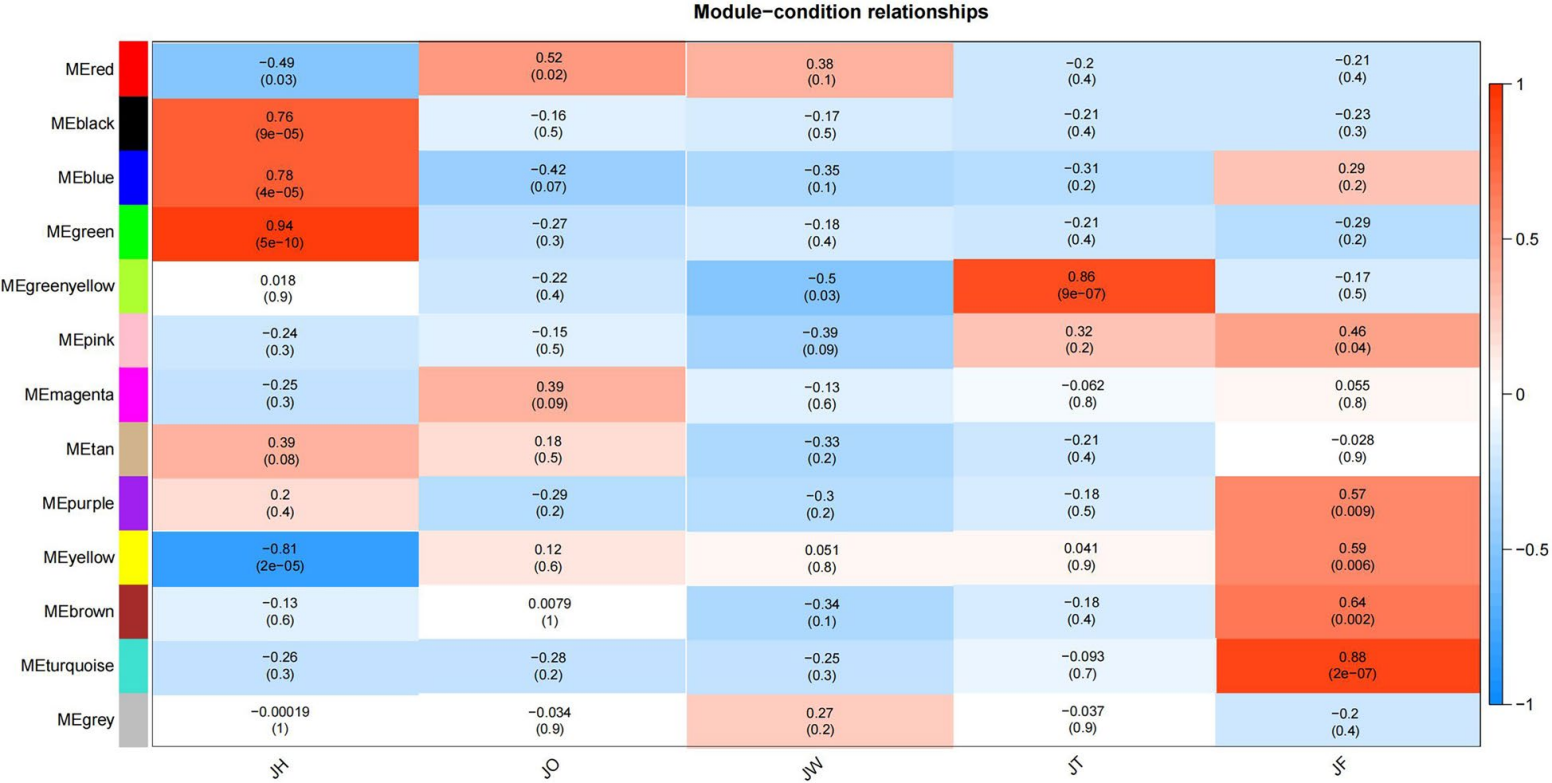
Module-condition relationships

**Fig. 8:**
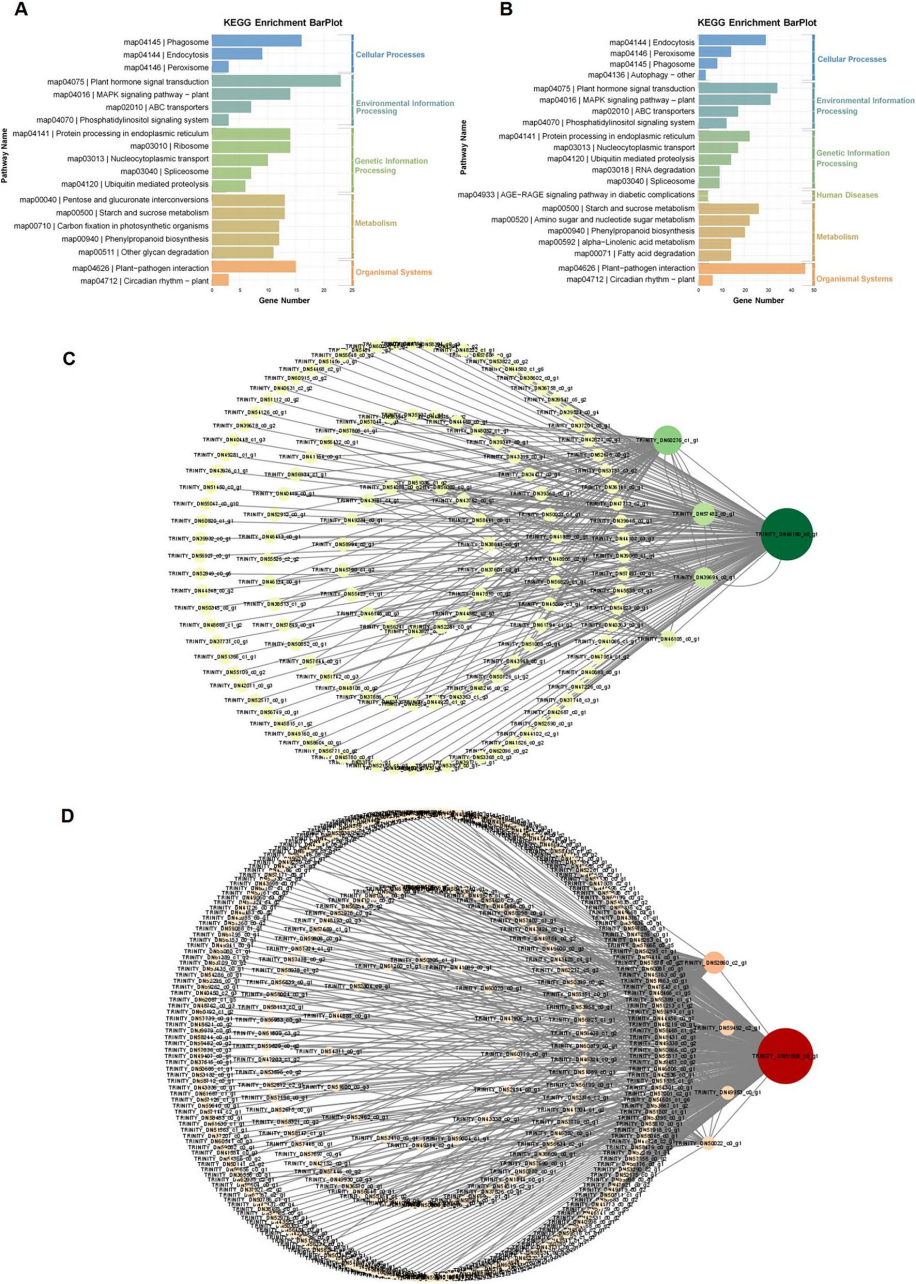
WGCNA. **A** KEGG enrichment of green module. **B** KEGG enrichment of yellow module. **C** Visualization of core genes of green module. **D** Visualization of core genes of yellow module

Removing genes with a low expression level, 21,194 genes were selected for WGCNA, resulting in the construction of 13 co-expression modules. In this study, modules with |*r*|> 0.80 and *p* < 0.001 were selected for further analysis. The green, green-yellow and turquoise modules were significantly correlated with disease traits: the green (*r* = 0.94, *p* = 5e-10) module displayed a significant positive correlation with disease traits in JH and negative correlation with disease defense traits in JO, JW, JT and JF; the greenyellow (*r* = 0.86, *p* = 9e-07) module showed a significant positive correlation with disease traits in JT; the turquoise (*r* = 0.88, *p* = 2e-07) module showed a significant positive correlation with disease defense traits in JF. The yellow (*r* = -0.81, *p* = 2e-05) module showed a very significant negative correlation in JH, and a positive correlation in JO, JW, JT and JF (Fig. 7). KEGG enrichment analysis of DEGs in green and yellow modules revealed that the enriched pathways mainly included plant hormone signaling transduction, MAPK signaling pathway, and plant-pathogen interactions (Fig. 8A, B). Therefore, these two modules might be the key modules involved in responding to ADM.

The top five genes with the highest KME (eigengene connectivity) values in each module were used as hub genes. In the green module, a total of 263 linear pairs were obtained with weight ≥ 0.3 as the threshold (Table S13). Among them, TRINITY_DN46180_c0_g1 (vesicle-associated protein 4–1, PVA41) was highly co-expressed with 141 genes. Among them 4 genes annotated to plant hormone signal transduction pathway, which were down-regulated at all disease stages (Fig. 8C, Table S14). In the yellow module, TRINITY_DN51806_c0_g1 (ubiquitin carboxyl-terminal hydrolase 16, UBP16), TRINITY_DN52860_c2_g1 (probable LRR receptor-like serine/threonine-protein kinase At5g10290, At5g10290), TRINITY_DN59492_c2_g1 (beta-fructofuranosidase, INV1), TRINITY_DN49953_c0_g1 (probable serine/threonine-protein kinase PBL2) and TRINITY_DN50022_c0_g1 (probable protein phosphatase 2C) were identified as hub genes. A total of 438 linear pairs were obtained with weight ≥ 0.25 as the threshold (Fig. 8D, Table S15). Among them, TRINITY_DN51806_c0_g1 was highly co-expressed with 274 genes, including 7 genes related to plant hormone signal transduction, 8 genes associated with the MAPK signaling pathway and 8 genes involved in plant-pathogen interactions. TRINITY_DN52860_c2_g1 and TRINITY_DN59492_c2_g1 responded to ABA, and co-expressed with genes involved in plant hormone signal transduction, the MAPK signaling pathway and plant-pathogen interactions (Table S16).

### qRT-PCR validation

**Fig. 9:**
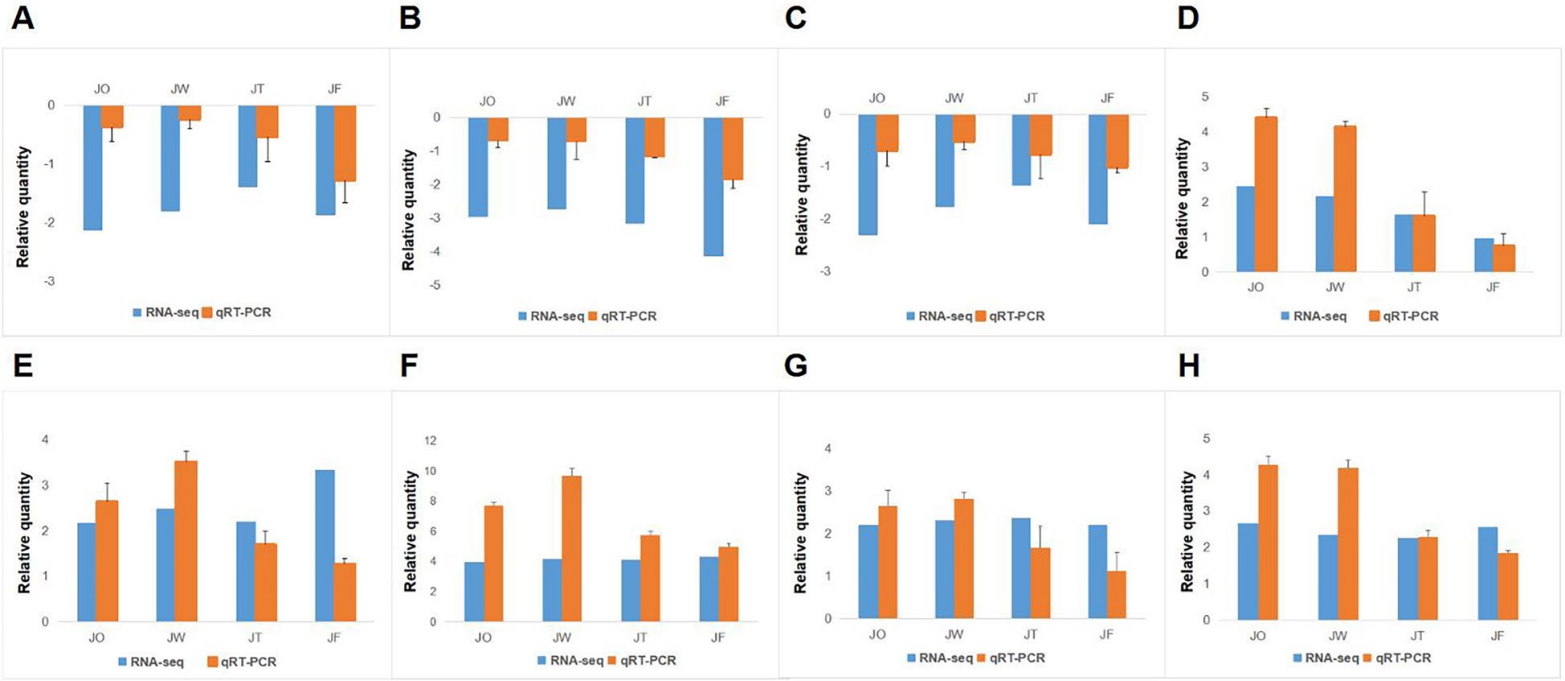
Relative quantity between qRT-PCR and RNA-seq from eight candidate genes. Each point represented a fold change value of expression at different disease group compared with that of control group. **A** AUX. **B** PYL. **C** BAK1. **D** JAZ. **E** MYC2. **F** EIX. **G** BZIP. **H** DLO2

To verify the accuracy of RNA-seq data, we verified the expression levels of 8 randomly selected DEGs by qR-TPCR. There is a consistent overall trend between RNA-Seq and qRT-PCR data (Fig. 9), which validates the reliability of RNA-seq data.

### ADM infection caused expression changes in major TF families

A total of 1,193 genes were predicted to be TFs. The three major TF families, sorted by the number of identified genes, were C2H2, bHLH and bZIP families. In total, 136 C2H2 genes were identified with multiple trends of expression (Table S17). 16 genes were up-regulated and 23 genes were down-regulated at all ADM stages, including 3 significantly down-regulated genes (TRINITY_DN41735_c0_g2, TRINITY_DN45943_c0_g3, TRINITY_DN54330_c0_g1). A total of 100 bHLH genes has been identified, each exhibiting a unique pattern of expression (Table S17), in which 18 genes were up-regulated and 36 genes were down-regulated at all ADM stages, including 4 significantly up-regulated genes (TRINITY_DN42615_c0_g3, TRINITY_DN47291_c0_g1, TRINITY_DN53877_c0_g1, TRINITY_DN56395_c0_g1) and 3 significantly down-regulated genes (TRINITY_DN39917_c0_g1, TRINITY_DN52661_c0_g1, TRINITY_DN54800_c0_g4). Similarly, there were 87 bZIP genes identified with contrasting trends of expression (Table S17). In total, 16 genes were up-regulated and 17 genes were down-regulated across all ADM stages. Among these, 2 genes were significantly up-regulated (TRINITY_DN32532_c1_g1, TRINITY_DN37463_c0_g1), while 2 others were significantly down-regulated (TRINITY_DN38074_c0_g3, TRINITY_DN38672_c0_g1).

Moreover, WRKY TFs are often associated with plant disease resistance. In our study, we identified 50 WRKY genes that exhibited contrasting trends of expression. 30 genes were up-regulated and 17 genes were down-regulated at all ADM stages. Among this, 6 significantly up-regulated genes (TRINITY_DN41303_c0_g1, TRINITY_DN48289_c0_g1, TRINITY_DN52457_c0_g1, TRINITY_DN52457_c0_g3, TRINITY_DN55902_c4_g1, TRINITY_DN60274_c0_g3) showed a strong correlation with pathogenesis (Table S17).

## Discussion

### Plant hormones changes

The dynamic endogenous hormones play an important role in plant-pathogen interactions. The contents of IAA, ABA, SA and JA in *A. carmichaelii* leaves were changed significantly at four ADM stages. Compared ADM stage 0, these four hormones were all lower at disease stage 1, and their contents significantly increased at disease stage 2. It proved that the endogenous hormones were disordered after *P. aconiti* invasion. The hormone contents initially decreased to appear *A. carmichaelii* infected symptoms, and later the restored hormone contents may have been due to the activation of the immune response. The decrease of IAA and ABA at stages 3 and 4 of ADM suggests that the synthesis of these compounds is inhibited during the later stages and that the inhibitory effect is more significant in ABA synthesis. The ABA and IAA response pathways interact synergistically or antagonistically to regulate plant development [26], the same trend of IAA and ABA content shows that they may synergistically work in response to ADM. IAA and ABA are often associated with plant susceptibility [27, 28], and we thought that IAA and ABA negatively regulate the responses to ADM in *A. carmichaelii*. Differently, the trend in SA and JA content in ADM stage 3 and 4 was opposite, so the SA and JA may have an antagonistic effect at a later stage, which is consistent with most studies on SA/JA. This could be that pathogens interfere with the phytohormone-mediated defense mechanisms, resulting in a decrease in hormone synthesis. In addition, hormone content was at its peak in ADM stage 2 and then gradually decreased, possibly due to the opening or closing of resistance channels. Phytohormones can regulate the response mechanism in plant and act as the crucial signaling molecules for activating disease responses when plant diseases occur [29, 30]. Huang et al. [31] showed that black-streaked dwarf virus infected rice by disrupting the balance of plant endogenous phytohormones, and that exogenous GA3 could alleviate the typical dwarfing symptoms in rice. Therefore, it is potential to use plant exogenous hormones relieve ADM in the next research.

Genes participated in the plant hormone biosynthesis pathway are closely related to both content and host defense. After ADM was introduced, the expression levels of genes related to phytohormone biosynthesis were also significantly regulated. Converting 9-cis-violaxanthin and 9-cis-neoxanthin into cis-xanthoxin, NCED is the rate-limiting enzyme for ABA biosynthesis, which affects ABA accumulation [32]. Due to the biosynthesis of JA, OsNCED3 played a positive role in defending against the brown planthopper of rice [33]. Our results suggested that the ABA content initially decreased with significantly down-regulated NCED genes. In *Luffa cylindrica,* JA, ABA and SA were overproduced along with increased expression of LOX2S, ERF, NCED2 and PAL genes due to inoculation with endophytic fungi. Rauf M et al. [34] verified this ultimately led to an increase in systemic immunity against downy mildew. The PALs were primarily up-regulated at all disease stages. We regard the NCED and PAL genes not only directly affect phytohormone biosynthesis, but also serve as both negative and positive regulators in response to ADM.

### Analysis of DEGs involved in hormone signaling

Plant hormones are vital endogenous signals that mediate complex signaling to resist diseases. For example, the AUX/IAA protein GhIAA43 was a negative regulator of cotton immune response during the infection of *Verticillium dahliae* [35]. Phytochrome A and phytochrome B in *Nicotiana tabacum* positively regulated plant defense responses to Chilli veinal mottle virus infection, and the contents of endogenous SA and JA and the expression of hormones signaling were lower in phytochromes mutant plants compared with wild type plants [36]. In our study, the DEGs in AUX, CTK, and GA signaling pathways were mostly significantly down-regulated at all disease stages. Most of the DEGs in ABA, ET, SA signaling pathways were significantly up-regulated. In the ABA signal transduction pathway, the ABA receptors, such as PYL, were mainly down-regulated. We infer that the signal transduction pathways of ET and SA are major components of the immune response mechanism of *A. carmichaelii* to ADM.

SA is an important plant hormone for establishing connections between diverse pathogens and plant diseases [37]. Systemic acquired resistance (SAR) is the most effective method to defend against pathogen infection. The pathogenesis-related protein PR can be induced to activate SAR after pathogens invasion. TGA TFs directly bind to multiple disease-related gene promoters to initiate the expression of disease resistance genes in SAR [38]. In our study, the DEGs encoding PR1 genes such as TRINITY_DN52956_c0_g1 and TRINITY_DN52956_c0_g2 were predominantly up-regulated, and the TGA gene (TRINITY_DN37463_c0_g1) showed significant up-regulation. This confirmed there is the presence of SAR immune responses in our study. Previous studies have indicated that CTR1 and MPK6 could regulate disease responses to prevent pathogen invasion. The silenced CTR1 tomato enhanced tolerance against tomato leaf curl virus infection [39]. MPK3/MPK6-mediated phosphorylation of ERF72 positively regulated the resistance of *Arabidopsis* to *Botrytis cinerea* [40]. The genes CTR1 and MPK6 were up-regulated at four disease stages, and this might have a positive effect on the disease defense of *A. carmichaelii.*

### Disease response/defense pathways and related genes

Notably, ADM affects metabolic processes such as plant-pathogen interactions, plant MAPK signaling pathways, and phenylpropanoid biosynthesis. Plants rely on PAMP-triggered immunity (PTI) and effector-triggered immunity (ETI) to detect invading pathogens and subsequently activate defense mechanisms [41]. Activation of Ca^2+^ signaling is a universal response to stress. For example, the CDPK gene regulates a broad-spectrum resistance to *Xanthomonas oryzae* in oryzae [42], and CML8 also work in *Pseudomonas syringae* plant immunity [43]. In the fungal PAMP pathway of our research, most genes encoding CDPK and CML were up-regulated. In the plant MAPK signaling pathway, the DEGs including MPK3, MPK6, WRKY22, ER and PDF1.2, were mostly up-regulated. The activation of these genes would lead to plant programmed cell death and pathogen defense responses. The phenylpropanoid biosynthesis pathway is the main synthetic pathway for many secondary metabolites, which plays an important role in responding to plant diseases. PAL is the first enzyme in phenylpropanoid metabolism and affects the accumulation of SA and phenylalanine-derived specialized metabolites [44]. PAL genes are the positive regulators of the SA-mediated defense pathway that helps combat aphid pests in sorghum [45]. The PAL1 gene increased resistance to the Cassava brown streak virus in cassava [46]. There were four significantly up-regulated PAL DEGs (TRINITY_DN41665_c2_g4, TRINITY_DN41931_c1_g1, TRINITY_DN41931_c1_g3, TRINITY_DN49908_c0_g1), which participated in the response to ADM at all disease stages. They could potentially be beneficial for disease resistance in *A. carmichaelii*.

In summary, several key defense genes involved in plant hormone signal transduction, plant-pathogen interaction, MAPK signaling pathway and phenylpropanoid synthesis pathways, are regulated under ADM. All above pathways and genes could potentially be responsible for either mediating or activating immune responses in *A. carmichaelii*. Our research also suggested that defensive responses could be significantly induced during the early stages of ADM, and most immune responses persisted throughout various stages of the disease. Furthermore, the host exhibited more diversified defensive responses at the later stage of ADM.

### Candidate genes by WGCNA

A total of 2 disease specific-modules (green and yellow) were identified through WGCNA. The enrichment analysis of green and yellow modules revealed that plant hormone signal transduction, plant-pathogen interactions and MAPK signaling pathways may participate in defense responses against ADM. Similarly, the genes in these two modules were also critical for responding to ADM.

The hub genes and genes with high connectivity to the hub genes were selected, and they may work on the response of *A. carmichaelii* to ADM. In green module, the hub gene TRINITY_DN57482_c0_g1 encoded an aspartic protease. Li C et al. [47] reported that the accumulation of the cotton aspartic protease GhAP1 increased the susceptibility of cotton plants to *V. dahliae*. Figueiredo L et al. [48] hold that 5 aspartic protease genes may be closely related to the tolerance to *P. viticola* in grapevine (*Vitis vinifea*)*.* The hub gene TRINITY_DN50022_c0_g1 encoded a protein phosphatase 2C (PP2C) in yellow module. Previous research showed that the PP2C regulates defense responses in *Arabidopsis* infected by *P. syringae* [49]. Meanwhile, SlPP2C genes were contributed to the resistance of tomato to bacterial wilt [50]. Therefore, TRINITY_DN57482_c0_g1 and TRINITY_DN50022_c0_g1 might regulate defense responses to ADM.

BKI1 is a receptor inhibitor of the BR and ERECTA (ER) signaling pathways. *Plasmopara viticola* effector PvRXLR131 suppresses plant immunity by targeting BKI1 [51]. The C4 protein of tomato leaf curl Yunnan virus has been confirmed to interact with BKI1 in tobacco. This interaction inhibits the dissociation of the ER-BKI1 complex, thus disrupting the activation of MAPK cascades. As a result, this leads to disease infection [52]. The TRINITY_DN38602_c0_g1 (BKI1) and TRINITY_DN49281_c1_g1 (BRI1) in green module have been annotated to plant hormone signal transduction pathway. The ubiquitylation of PSKR1 regulates the defense response to *B. cinerea* in tomato [53]. OsPSKR1 regulates resistance to *X. oryzae* by activating the pathogenesis-related genes in the SA pathway in rice. In yellow module, TRINITY_DN48431_c0_g1 encoding the phytosulfokine receptor 1 (PSKR1) was a highly connected gene with TRINITY_DN51806_c0_g1 and was annotated to plant hormone signal transduction and MAPK signaling pathways. In addition, the analysis of DEGs showed that TRINITY_DN38602_c0_g1, TRINITY_DN49281_c1_g1, and TRINITY_DN48431_c0_g1 were significantly regulated, and they could potentially respond to ADM.

### The role of TFs

TFs could regulate the life activities of plants during their growth and play a key regulatory role under pathogenic stress. TFs such as C2H2 [54], bHLH [55], bZIP [56] and WRKY [57] were associated with the immune response of plants to pathogen infection. The TFs that were differently expressed at all disease stages, such as C2H2, bHLH and bZIP, may play an important role as regulatory factors in *A. carmichaelii*’s response to ADM. Meanwhile, WRKY TFs are key regulators of plant immunity. An increased expression in WRKY TFs suggests that they may positively regulate the resistance to ADM in *A. carmichaelii*.

## Conclusions

ADM infestation in *A. carmichaelii* leaves can produce complex responses. As the disease developed, the level of endogenous plant hormones was changed, and the expression of related genes was induced. GO annotation and KEGG enrichment analysis showed that the pathways that were highly regulated in response to ADM included plant hormone signal transduction, plant-pathogen interaction, MAPK signaling pathway-plant and phenylpropanoid synthesis pathways. Combining with WGCNA, we have further analyzed the pathways and genes associated with resistance responses. We have also reported a large number of candidate genes related to immune responses. Overall, the results described here improved our understanding of molecular responses to ADM and have contributed to the development of strategies for combating downy mildew during the cultivating *A. carmichaelii.*

### Supplementary Information

**Supplementary Material 1.**

**Supplementary Material 2.**

**Supplementary Material 3.**

**Supplementary Material 4.**

## Data Availability

The *Aconitum carmichaelii* leaves used in this experiment were permitted to been collect from private land. The raw sequencing reads of transcriptome data have been deposited to the NCBI Sequence Read Archive (SRA) as Bioproject PRJNA1046272 (https://www.ncbi.nlm.nih.gov/sra/PRJNA1046272).

## Abbreviations

ADM: *Aconitum carmichaelii* Debx downy mildew
RNA-seq: RNA sequencing
DEGs: Differentially expressed genes
WGCNA: Weighted gene co-expression network analysis
IAA: Indole-3-acetic acid
ABA: Abscisic acid
SA: Salicylic acid
JA: Jasmonic acid
ELISA: Enzyme-Linked ImmunoSorbent Assay
GO: Gene ontology
TPM: Transcripts per kilobase million
qRT-PCR: Real-time quantitative polymerase chain reaction
EIF: Eukaryotic initiation factor
TF: Transcription factor
MAPK: Mitogen-activated protein kinase
CC: Cellular component
BP: Biological processe
MF: Molecular function
CDPK: Calcium-dependent protein kinases
RBOH: Respiratory burst oxidase homologue
CAM: Calmodulin
CML: Calcium-binding proteins
WRKY1: WRKY transcription factor 1
ET: Ethylene
PDF 1.2: Defensin-like protein 16
PAL: Phenylalanine ammonia lyase
AUX: Auxin
TAA1: L-tryptophan aminotransferase of *Arabidopsis* 1
YUC: Indole-3-pyruvate monooxygenase YUCCA
DDC: Aromatic-L-amino-acid decarboxylase
ALDH: Aldehyde dehydrogenase
ALDH7A1: Aldehydede hydrogenase family 7 member A1
ZEP: Zeaxanthin epoxidase
NCED: 9-Cis-epoxycarotenoid dioxygenase
XanDH: Xanthoxin dehydrogenase
AAO3: Abscisic-aldehyde oxidase 3
ABA2: Xanthoxin dehydrogenase
CYP707A: ABA 8'-hydroxylase CYP707A
AOG: Abscisate beta-glucosyltransferase
PLA2: Phospholipase A2
PLA1: Phospholipase A1
TGL4: Triacylglycerol lipase TGL4
LOX: Lipoxygenase
AOS: Hydroperoxide dehydratase
AOC: Allene oxide cyclase
OPR: 12-Oxophytodienoic acid reductase
OPCL1: OPC-8:0 CoA ligase 1
ACX: Acyl-CoA oxidase
CTK: Cytokinin
GA: Gibberellin
BR: Brassionosteroid
AUX/IAA: AUX-responsive protein AUX/IAA
ARF: AUX-responsive factor ARF
TIR1: Transport inhibitor response 1
SAUR: SAUR family protein
GH3: AUX-responsive GH3
HP: Histidine-containing phosphotransfer peotein
SnRK2: Serine/threonine-protein kinase SnRK2
ERF1: ET-responsive transcription factor 1
CTR1: Constitutive triple response 1
MPK6: Mitogen-activated protein kinase 6
PVA41: Vesicle-associated protein 4–1
UBP16: Biquitin carboxyl-terminal hydrolase 16
At5g10290: LRR receptor-like serine/threonine-protein kinase At5g10290
INV1: Beta-fructofuranosidase
SAR: Systemic acquired resistance
PTI: PAMP-triggered immunity
ETI: Effector-triggered immunity
