# Supplementary figures and images for: Comparative transcriptome revealed the molecular responses of Aconitum carmichaelii Debx. to downy mildew at different stages of disease development
Source: BMC Plant Biol. 2024 Apr 25;24:332. doi: 10.1186/s12870-024-05048-x (PMC11044490; doi:10.1186/s12870-024-05048-x)

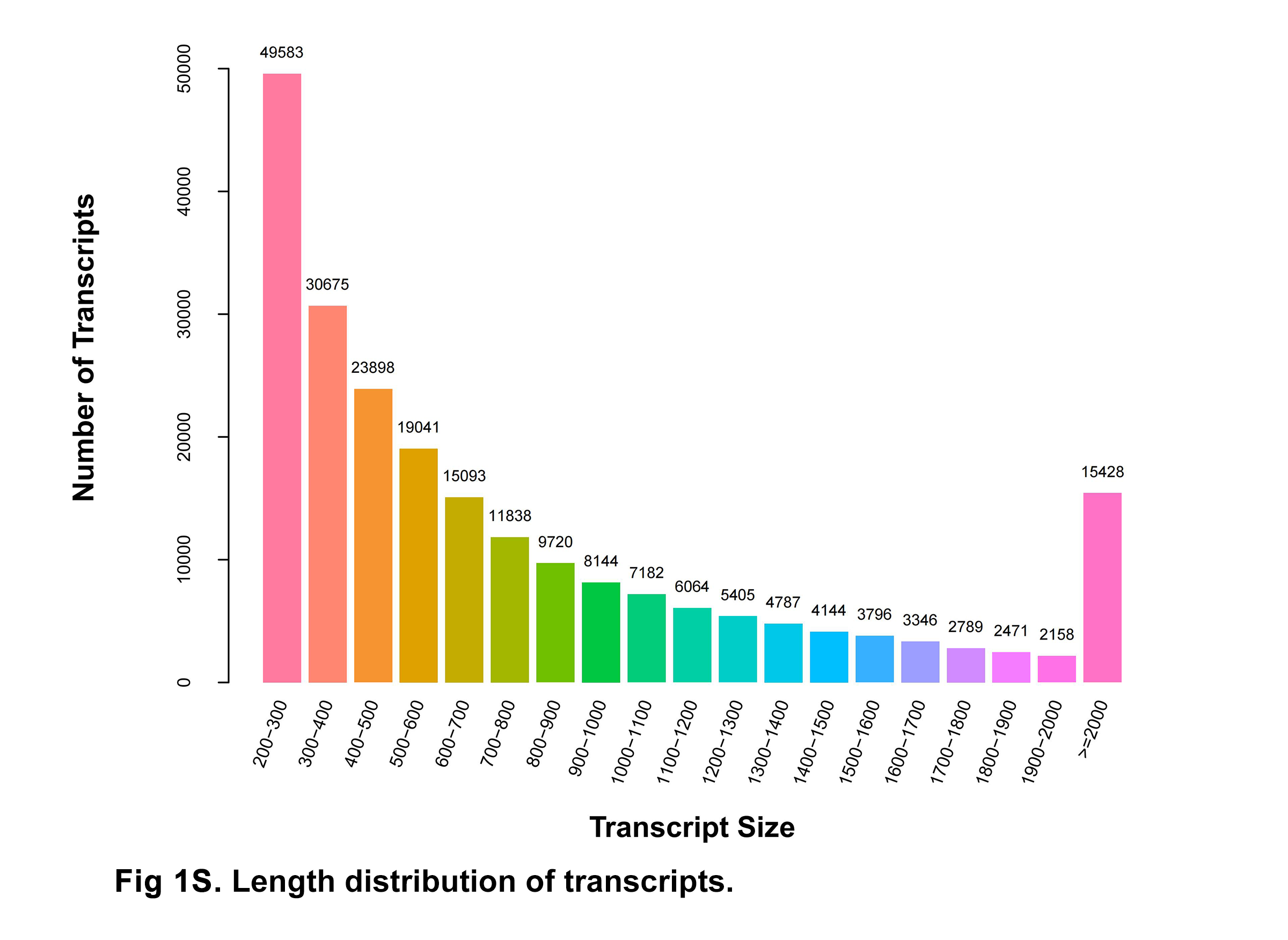

Supplement: Supplementary file 2 — Supplementary Material 2. [file 12870_2024_5048_MOESM2_ESM.tif]

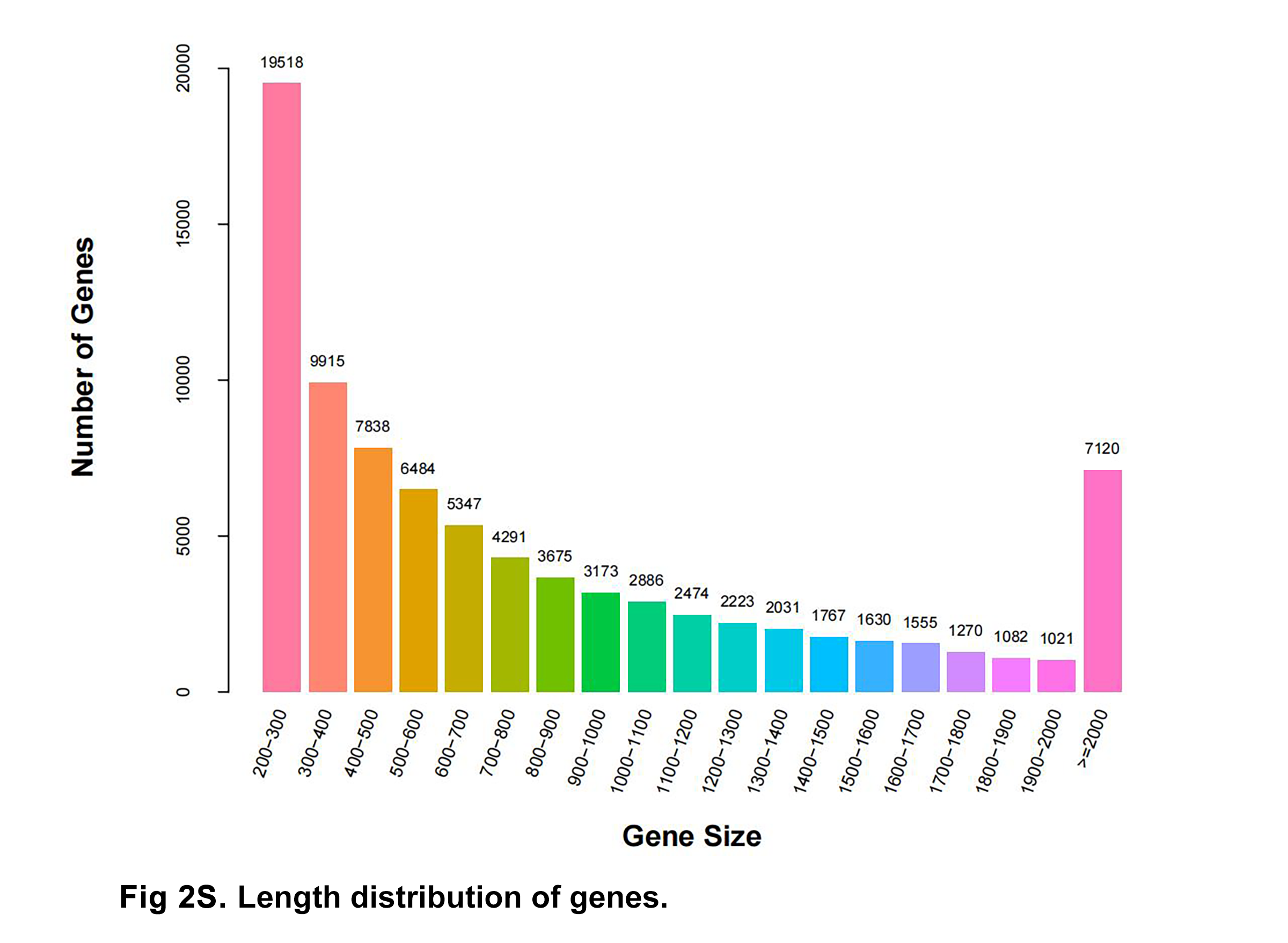

Supplement: Supplementary file 3 — Supplementary Material 3. [file 12870_2024_5048_MOESM3_ESM.tif]

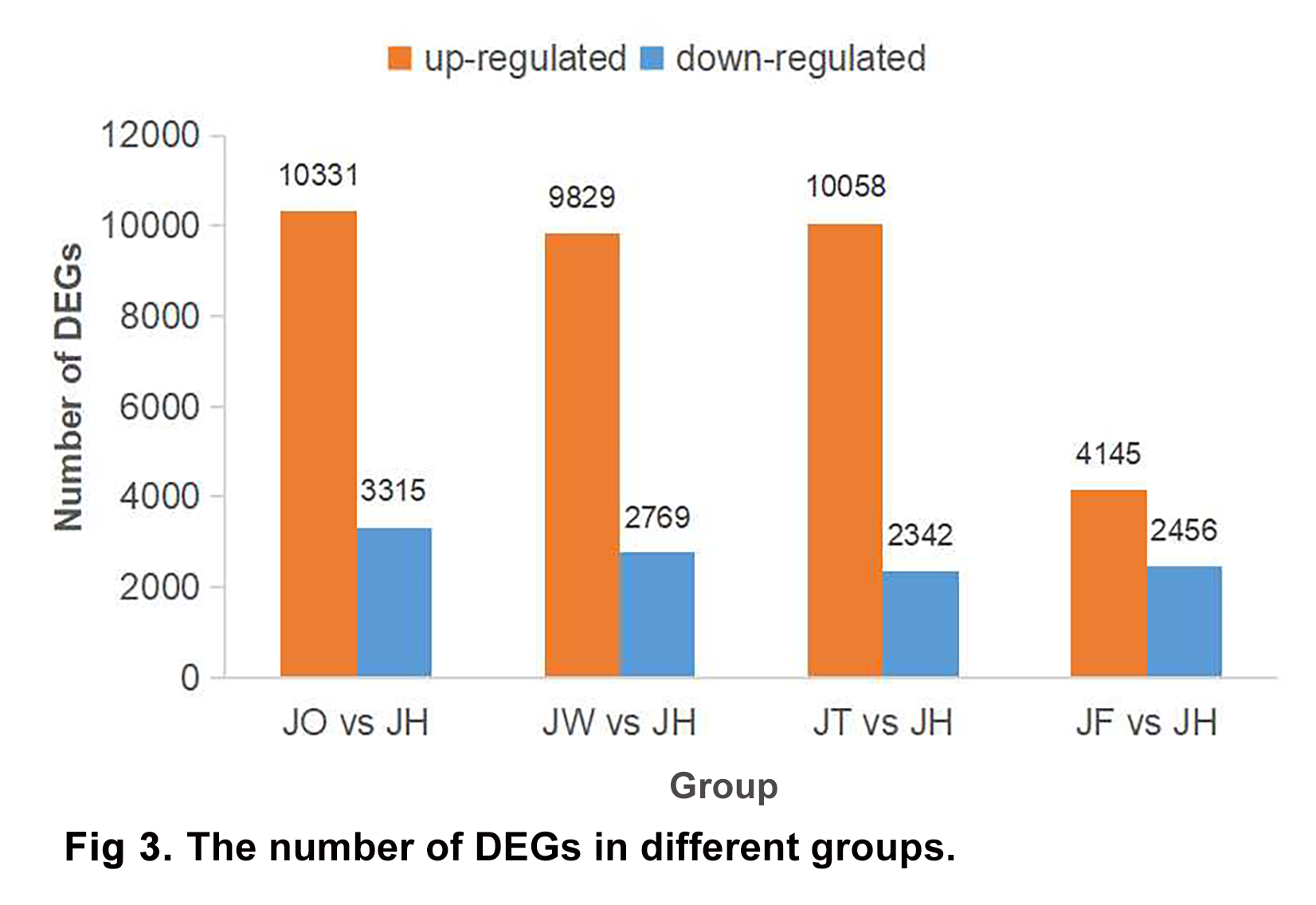

Supplement: Supplementary file 4 — Supplementary Material 4. [file 12870_2024_5048_MOESM4_ESM.tif]
